# The SCOC database, a large, open, and global database with sediment community oxygen consumption rates

**DOI:** 10.1038/s41597-019-0259-3

**Published:** 2019-10-29

**Authors:** Tanja Stratmann, Karline Soetaert, Chih-Lin Wei, Yu-Shih Lin, Dick van Oevelen

**Affiliations:** 1 NIOZ Royal Netherlands Institute for Sea Research, Department of Estuarine and Delta Systems, and Utrecht University, P.O. Box 140, 4400 AC Yerseke, The Netherlands; 2 Marine Biology Research Group, Ghent University, Krijgslaan, 281 S8, 9000 Ghent Belgium; 3 Present Address: Department of Earth Sciences, Utrecht University, Vening Meineszgebouw A, Princetonlaan 8a, 3584 CB, Utrecht, The Netherlands; 4 Institute of Oceanography, National Taiwan University, No. 1, Sec. 4, Roosevelt Road, Taipei, 105 Taiwan; 5 Department of Oceanography, National Sun Yet-san University, 70 Lienhai Rd., Kaohsiung, 80424 Taiwan

**Keywords:** Marine biology, Carbon cycle

## Abstract

Sediment community oxygen consumption (SCOC) rates provide important information about biogeochemical processes in marine sediments and the activity of benthic microorganisms and fauna. Therefore, several databases of SCOC data have been compiled since the mid-1990s. However, these earlier databases contained much less data records and were not freely available. Additionally, the databases were not transparent in their selection procedure, so that other researchers could not assess the quality of the data. Here, we present the largest, best documented, and freely available database of SCOC data compiled to date. The database is comprised of 3,540 georeferenced SCOC records from 230 studies that were selected following the procedure for systematic reviews and meta-analyses. Each data record states whether the oxygen consumption was measured *ex situ* or *in situ*, as total oxygen uptake, diffusive or advective oxygen uptake, and which measurement device was used. The database will be curated and updated annually to secure and maintain an up-to-date global database of SCOC data.

## Abstract (summary)

**Table:** 

Measurement(s)	Oxygen Consumption
Technology Type(s)	digital curation • incubation
Factor Type(s)	geographic location • depth • continental slope • continental rise • abyssal plain • near-shore area • continental shelf
Sample Characteristic - Environment	sediment
Sample Characteristic - Location	Mediterranean Sea • Southern Ocean • Atlantic Ocean • Pacific Ocean • Arctic Ocean • Indian Ocean • Gulf of Mexico • Black Sea • Red Sea

Machine-accessible metadata file describing the reported data: 10.6084/m9.figshare.9960131

## Background & Summary

Marine sediments play a key role in the global carbon cycle^1^. Organic matter either deposits on sediments as detritus or marine snow, or is produced by phototrophic and chemo-autotrophic organisms. Part of the organic matter is buried on geological time scales, but the largest share is remineralized by abundant prokaryotes, protozoans, and metazoans that are predominantly active in the top 10 to 50 cm of the sediment. Sediment community oxygen consumption (SCOC) is a generally accepted proxy for total organic matter degradation in the marine sediments as it integrates degradation through aerobic activity, autotrophic and heterotrophic nitrification, *and* re-oxidation of reduced inorganic compounds that were generated during anaerobic heterotrophic degradation of organic matter^2–4^. In abyssal sediments, aerobic respiration dominates SCOC (76% of SCOC^5^), whereas in coastal areas, re-oxidation processes contribute most to oxygen consumption (52 to 76% of SCOC^6^). Hence, SCOC is considered a useful proxy for total organic carbon mineralization and it has been used to derive respiration rates of the deep (>200 m water depth) global seafloor^7^, to calculate the flux of organic carbon to the seafloor^8^ and to determine benthos-mediated turnover of carbon at the seafloor^9^.

The general validity of SCOC has initiated the development of global SCOC databases since the mid-1990s^7–9^ consisting respectively of 136^8^ and 490^7^ SCOC measurements taken from 4 m (near-shore areas) to 5,200 m (abyssal plains) water depth and cover the Atlantic Ocean including the Mediterranean Sea, the Pacific Ocean, and the Indian Ocean. The most recent database of global SCOC measurements from 2018^9^ comprises 1,075 data points from all ocean basins and the Mediterranean Sea.

In all three cases, however, the database compilation procedure was not transparently described and hence it remains unclear which studies were included in the databases and which exclusion criteria were applied. More importantly even, the actual SCOC values and supporting information, such as geographic location or measurement approach, were not presented or otherwise made available. Hence, the reader cannot judge which studies were omitted, whether low quality data were incorporated and supplement the existing database with new data.

Here, we present the publicly available ‘SCOC database’^10^ that contains a list of 315 studies that were identified following the procedure for systematic reviews and meta-analyses^11^. This standardized procedure provided a dataset with 3,540 georeferenced SCOC records (in mmol O_2_ m^−2^ d^−1^) that were extracted from 230 selected studies. All records include information about how the measurements were taken in the original studies so that researchers can compare novel SCOC data with entries in the database. The SCOC database also allows researchers to select data that were collected with specific techniques for further analyses and it shows which areas in the world are under-sampled and, in this way, may stimulate future sampling campaigns.

## Methods

**Fig. 1:**
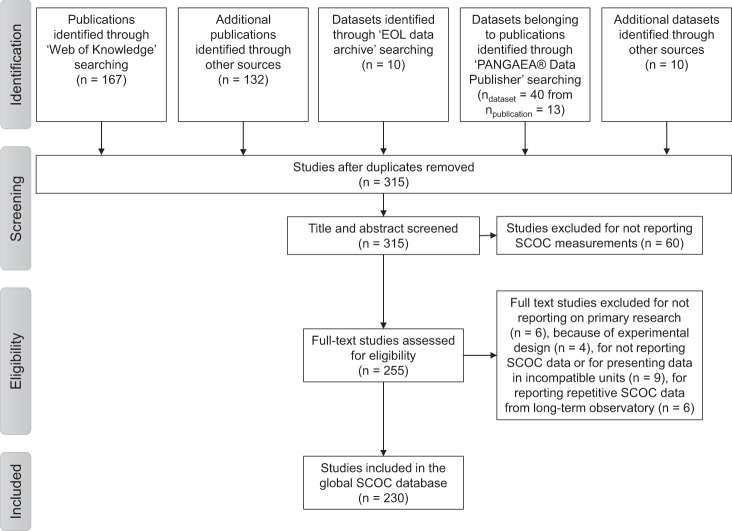
Flow chart showing the database compilation procedure. It describes how publications and datasets were identified and which selection criteria were applied to exclude studies from the final SCOC database.

The SCOC database was compiled in April 2019 following the PRISMA (“Preferred Reporting Items for Systematic reviews and Meta-Analyses”) Statement for systematic reviews and meta-analyses^11^ (Fig. 1). In the first phase of the systematic review (Identification), 167 publications were identified in the *Web of Science* using the key words “sediment community oxygen consumption”, “marine benthic respiration” and “benthic oxygen demand”. Additional 132 publications were identified through expert knowledge and examining literature cited in review papers. Using the keywords “sediment respiration oxygen uptake rates”, “benthic respiration”, “SCOC”, “sediment oxygen flux”, “sediment community oxygen consumption”, and “benthic oxygen demand”, 50 datasets were found in the *EOL data archive* (http://data.eol.ucar.edu/) and the *PANGAEA® Data Publisher* (https://www.pangaea.de/). Further six datasets from the *PANGAEA® Data Publisher* and the *EOL data archive* were found through citations in a review paper and four datasets included unpublished SCOC measurements. After removing duplicates, the titles and abstracts of 315 studies (Table 1) were screened in the Screening phase during which 60 studies were excluded because they did not report marine SCOC measurements. Of these excluded studies, two studies were palaeoceanographic studies, four studies investigated biogeochemical cycling of other chemical elements (e.g., S, N, Hg), seven studies dealt with the pelagic realm, and one study was about physical oceanography. Furthermore, eleven studies were freshwater studies, ten studies presented models and simulations, nine studies dealt with research about specific (individual) species, two studies were experimental (manipulative) studies, for studies introduced new methods and/or new research instrumentation, two studies were review papers or meta-analyses, three studies reported benthic biomasses or abundances, four studies analyzed specific compounds in the sediment (e.g. total organic carbon, pigments), and one study measured oxygen consumption of a coral reef, but not of the sediment community. In the Eligibility phase, 255 studies were examined of which 25 were excluded because they did not report SCOC data or because of the experimental design of the study. Further reasons for removing studies were the presentation of SCOC data in units that could not be converted to mmol O_2_ m^−2^ d^−1^. Moreover, studies were excluded when they did not report primary research, i.e., original measurements, or when they presented the same measurements from a long-term observatory. The final SCOC database included 230 studies from which a total of 3,540 georeferenced SCOC entries were extracted (Table 1).

**Table 1:** Specification of the SCOC database with file locations.

Source	Document name	Number of studies (records)	Data description	Method
10.5061/dryad.25nd083	List of studies (version 1)	315	Alphabetical reference list of studies about sediment community oxygen consumption (SCOC) that were identified following the PRISMA Statement. It is further specified which studies were excluded during the screening process and eligibility assessment.	Literature search
10.5061/dryad.25nd083	SCOC database (version 1)	230 (3,540)	All SCOC records included in the SCOC database.	Extraction of SCOC records from literature

When studies did not indicate the exact sampling or measurement location in geographical coordinates (latitude, longitude), but only revealed the sampling area or presented it in maps, the coordinates of the specific location were approximated using *Google Maps*. When data were only presented as mean or median ± error term, only the mean and median, respectively, were included in the dataset. In cases where SCOC rates were reported in figures instead of tables, the rates were extracted from the figure using ImageJ^12^.

## Data Records

The SCOC database^10^ is publicly available on *Dryad Digital Repository* and consists of two csv.files, the *List of studies* file and the *SCOC database* file. The *List of studies* specifies alphabetically the references of all 315 studies that were identified during the Identification phase of the systematic review after duplicates were removed. For each data entry, the SCOC database file includes information about the region were the SCOC was measured and the corresponding ocean. The geographical location (latitude, longitude), water depth (in m), and the depth range classification according to Dunne et al. (near-shore area: 0–50 m water depth, continental shelf: >50–200 m water depth, continental slope: >200–2,000 m water depth, continental rise/abyssal plain: >2,000 m water depth)^13^ are reported. The database also states whether SCOC (in mmol O_2_ m^−2^ d^−1^) was measured *ex situ* or *in situ*, as total oxygen uptake (TOU)^2^, as diffusive oxygen uptake (DOU)^2^, or as advective oxygen uptake (AOU)^14^. In addition, the measurement techniques, i.e., benthic incubation chamber, core incubation, lander with benthic chambers, eddy covariance system, core microprofiling, lander with microprofilers, porewater extraction, and flow through reactors (Online-only Table 1), are tabulated. Information about sediment type and median grain size (in μm) are provided whenever possible, as well as information about whether sediments are photosynthetic and whether measurements were conducted in the dark or in the light. The SCOC database will be curated through Dryad and an update of the database with the inclusion of new studies will be created annually.

## Technical Validation

### Geographic and water depth bias

**Fig. 2:**
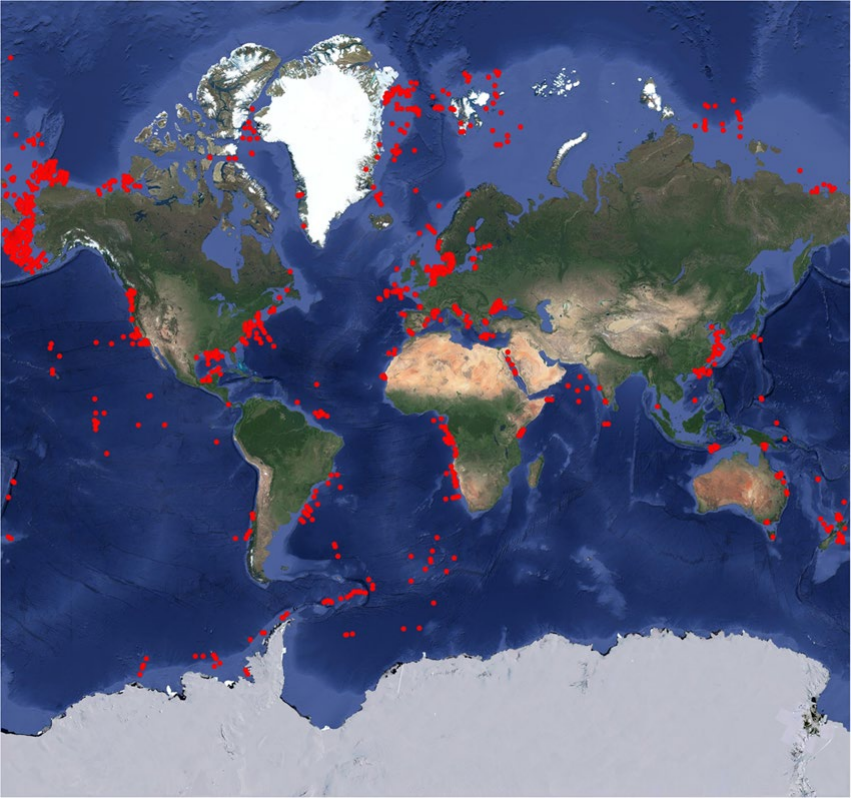
Global distribution of sampling stations where sediment community oxygen consumption was measured. Several dots show multiple measurements.

**Fig. 3:**
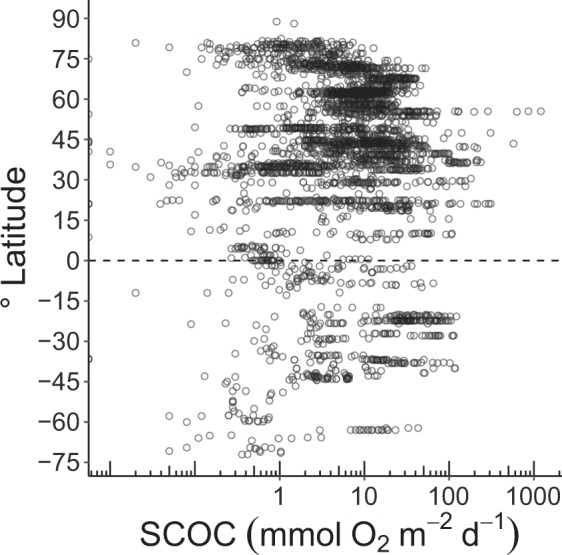
Sediment community oxygen consumption (mmol O_2_ m^−2^ d^−1^) along a latitudinal gradient. Each dot shows a single measurement and the dashed line indicates the equator. Notice the logarithmic scale on the x-axis.

Between 1968 and 2019, 41.3% of the SCOC measurements were taken in the Pacific Ocean, 35.1% were taken in the Atlantic Ocean (including the Gulf of Mexico and Mediterranean Sea), and 17.7% were taken in the Arctic Ocean (Fig. 2). Most of these measurements were performed in the northern hemisphere between 1.00°N and 88.8°N (84.9%) (Fig. 3). In contrast, only 14.3% of the SCOC data originate from the southern hemisphere between 1.00°S and 72.1°S (Fig. 3), and <1% of the measurements were taken within 1° around the equator (Fig. 3). Especially the Indian Ocean is severely under sampled (2.20% of all data entries; Fig. 2).

**Fig. 4:**
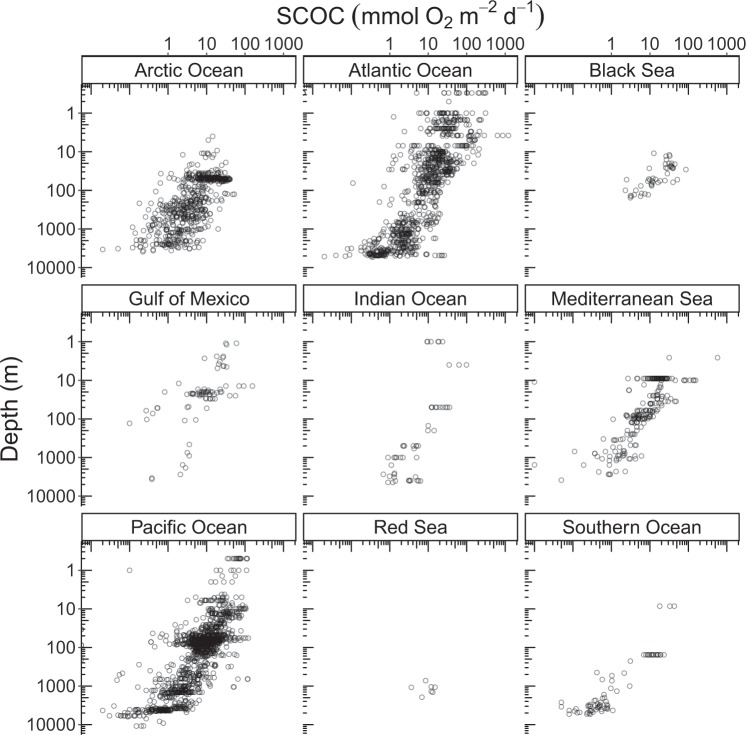
Sediment community oxygen consumption (mmol O_2_ m^−2^ d^−1^) along a water depth gradient (m). Notice the logarithmic scale on both axes.

Though near-shore areas account for only 2.03% of the global seafloor (7.10 × 10^12^ m^2,13^), 33.7% of all SCOC measurements were taken at ≤50 m water depth (Fig. 4). In contrast, the continental rises and abyssal plains cover 88.9% of the seafloor (3.50 × 10^14^ m^2,13^), but contributed only 14.1% of the data to the SCOC database (Fig. 4). Hence, the database is biased towards the shallow waters (≤200 m water depth) of the Pacific and Atlantic Ocean.

### Differences in sediment community oxygen consumption measurements

SCOC was quantified as TOU (80.5% of all measurements), as DOU (18.4% of all measurements), and as AOU (0.31% of all measurements). No information about measurement techniques was given for 0.56% of the measurements. As TOU is the sum of DOU, AOU, and benthos mediated oxygen uptake^2,14^, i.e. faunal respiration and irrigation, DOU and AOU are underestimates of the actual SCOC. Glud and colleagues^15^, for example, measured a 10.7 mmol O_2_ m^−2^ d^−1^ higher TOU rate compared to the DOU rate at a 1,747 m deep location in the southern Atlantic Ocean, where a dense population of foraminifera lived. Hence, it is recommended to use SCOC data measured as TOU at sites with high fauna abundance. When no fauna is present, e.g. in many deep-sea sediments, DOU equals TOU in cohesive, impermeable sediments and both values can be used as a proxy for total benthic carbon mineralization rate^2^. However, in permeable, sandy sediments that cover 47% of the continental shelf up to a water depth of <65 m^16^, oxygen uptake via advective porewater transport of oxygen can be more than 1.5 times larger than diffusive oxygen uptake^17^. This advective pore-water transport results from an interaction between bottom water currents and sediment topography^18^. Hence, SCOC measurement techniques that cut natural bottom flows off during sediment incubation, such as sediment core incubations or microprofiling of sediment cores, are not suited to measure SCOC in this type of sediment. Instead, the non-invasive eddy covariance technique should be used for medium to coarse grained sands^19^.

SCOC measurements can vary up to factor of 4 depending on whether they are measured *in situ* or *ex situ*^2^. At water depth >600 m, *ex situ* DOU measurements overestimate SCOC^2^, whereas *in situ* and *ex situ* measurements of SCOC only differ by a factor of 1.3 at 21 m water depth^20^. Therefore, it is recommended to use exclusively *in situ* SCOC measurements at >600 m water depth.

Photosynthesis in photic sediments strongly influences SCOC and leads to differing oxygenated sediment horizons with oxygen supersaturation below the sediment-water interphase and a longer oxygen penetration depth compared to aphotic sediments^2^. This should be considered when SCOC rates from phototrophic systems are used.

## Supplementary information

R markdown file.

## Data Availability

The R code used to prepare Figs 3 and 4 can be found in the electronic supplement.

## Online-only Table

**Online-only Table 1:** Short description of the various methods used to measure sediment community oxygen consumption (SCOC) as total oxygen uptake (TOU), diffusive oxygen uptake (DOU), and advective oxygen uptake (AOU).

Measurement technique	Type of SCOC	Description
Benthic incubation chamber	TOU	Chamber that is open at the bottom with a removable or permanently fixed gas-tight lid at the top. The lid can accommodate an oxygen optode for continuous recordings of oxygen concentration, and/or sampling ports to take water samples. To measure SCOC, the chamber is inserted several centimeters into the seafloor. In this way, the inside of the chamber is sealed off from the surrounding seawater and a change in oxygen concentration inside the chamber can be monitored over time.
Sediment core incubation	TOU	Recovered sediment cores that are filled with sediment and overlying seawater are closed with air-tight lids. During the incubation in the laboratory, the oxygen concentration in the overlying seawater in the sediment cores is monitored over time with an oxygen optode or electrode.
Lander with benthic chambers	TOU	Often a tripod that carries benthic incubation chambers. It is free-falling or can be lowered with a cable to the seafloor. At the seafloor, the incubation chambers are inserted several centimeters into the sediment and the oxygen concentration in the seawater inside the chamber is monitored with oxygen optodes. Depending on the design of the lander, the benthic chambers can be closed at the bottom to trap the sediment that has been incubated and lift it to the sea surface.
Eddy covariance system	TOU	The eddy covariance or correlation technique measures SCOC non-invasively. By simultaneously recording vertical water velocity with an acoustic doppler velocimeter 10 to 50 cm above the seafloor and measuring the fluctuation of oxygen concentration with oxygen microelectrodes at the same point, the flux of oxygen towards the sediment surface can be calculated^1^.
Core microprofiling	DOU	Vertical profiles of oxygen concentration in the sediment-water interphase and sediments of retrieved sediment cores are measured with micrometer-thick oxygen electrodes. Subsequently, DOU is calculated by linearly approximating the gradient in oxygen concentration in the diffusive boundary layer or below the sediment-water interphase using Fick’s first law of diffusion^2^.
Lander with microprofilers	DOU	Usually a tripod equipped with a set of oxygen microelectrodes that is free-falling or lowered to the seafloor at a cable. At the seafloor, the oxygen microelectrodes record vertical profiles of oxygen concentration in the sediment which are analyzed after lander retrieval.
Porewater extraction	DOU	Porewater from several depth intervals in the upper meters of sediment is collected with an *in situ* porewater sampler (e.g.^3^). Subsequently, oxygen concentration in the porewater samples is measured and DOU is calculated from the resulting vertical oxygen profiles using Fick’s first law of diffusion^4^.
Flow through reactor (FTR)	AOU	Flow through reactors are equipped with oxygen optodes or oxygen flow-through cells^5^ at the inlet and outlet of the FTR and filled with homogenized surface sediment. Seawater is pumped through the FTR and the oxygen concentration in the water is monitored at the inlet and the outlet. Volumetric oxygen consumption is calculated based on the difference in oxygen concentration between the two measuring points and the retention time^5^. Subsequently, SCOC is calculated based on the oxygen penetration depth in the natural sediment and the volumetric oxygen consumption^5^.
