# Supplementary material for: The SCOC database, a large, open, and global database with sediment community oxygen consumption rates
Source: Sci Data. 2019 Oct 29;6:242. doi: 10.1038/s41597-019-0259-3 (PMC6820755; doi:10.1038/s41597-019-0259-3)
Supplement: Supplementary file 1 — R markdown file. [file 41597_2019_259_MOESM1_ESM.pdf]

Global SCOC database

Tanja Stratmann

August 8, 2019

```
knitr::opts_chunk$set(echo = TRUE, warning=FALSE, message=FALSE)
```

Loading SCOC dataset

```
setwd("C:/Users/TanjaS/Documents/paper/Paper 3_Global dataset of SCOC/data an
alysis")
SCOC_data = read.csv("SCOC database.csv")  # read csv file

require(ggplot2)
require(scales)
require(varhandle)
require(scales)
require(psych)
require(scales)

SCOC_data <- SCOC_data[c(1:3540), c(2:4, 6:9, 11, 16)]

cols <- c("Ocean","Latitude", "Longitude", "Depth", "Depth_range", "SCOC", "e
x_in_situ","TOU_DOU", "Reference")
colnames(SCOC_data) <- cols
```

Preparing figure 3: SCOC along a latitudinal gradient

```
data_figure3 <- SCOC_data[, c(2, 6)]
data_figure3 <- na.omit(data_figure3)
data_figure3$Latitude <- as.numeric(data_figure3$Latitude)

ggplot(data = data_figure3, aes(x = SCOC, y = Latitude)) +
    geom_point(alpha = 0.5, size=1.5, shape=1) + theme_classic() +
    scale_y_continuous(breaks = c(-90, -75, -60, -45, -30, -15, 0,
                              15, 30, 45, 60, 75, 90)) +
  xlab(bquote(' '*SCOC~(mmol~O[2]~m^-2~d^-1*''))) + ylab("Â° Latitude") +
  theme(axis.text.x = element_text(vjust=0.5, size=15)) +
  theme(axis.text.y = element_text(vjust=0.5, size=15)) + scale_x_continuous(
trans=log_trans(10), breaks = c(1, 10, 100, 1000)) + annotation_logticks(side
s = "b") +
  theme(text = element_text(size=20)) + geom_hline(yintercept = 0, linetype="
dashed")+ theme(plot.margin=unit(c(0.5,0.5,0.5,0.5), "cm"))
```

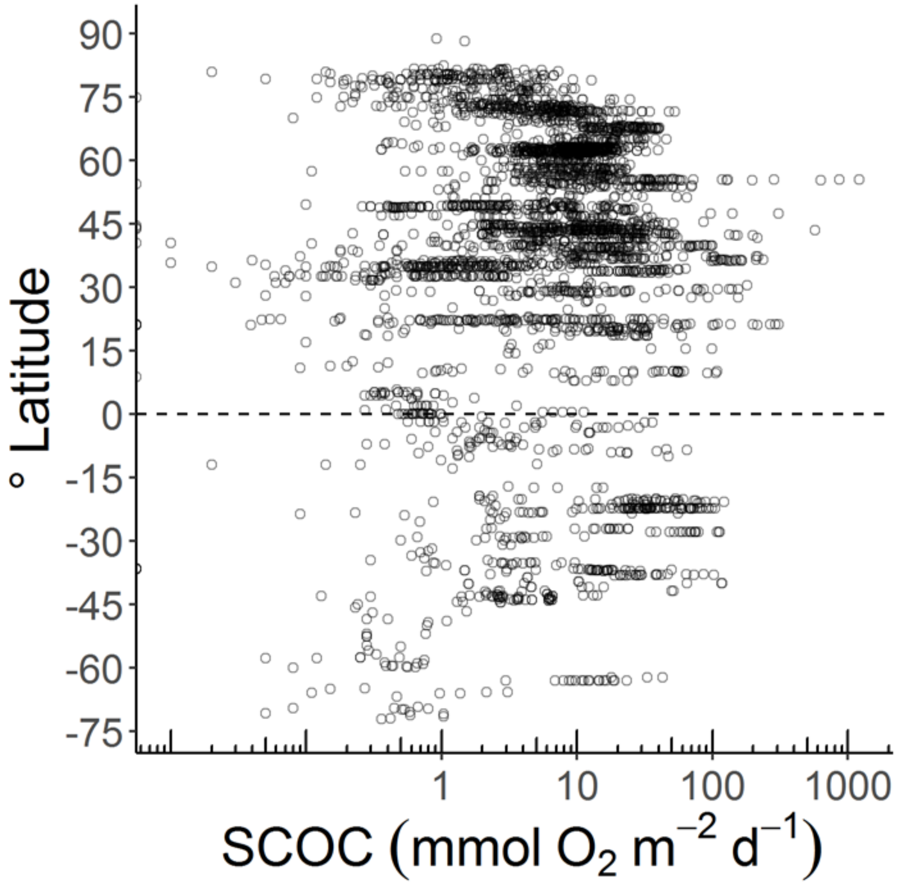


```
ggsave(file="Figure 3.png", width=5,height=5)
ggsave("Figure 3.pdf", width=5,height=5)
```

Preparing figure 4: SCOC along a depth gradient

```
data_figure4 <- SCOC_data[, c(1, 4, 6)]
data_figure4$Depth <- as.numeric(data_figure4$Depth)

reverselog_trans <- function(base = exp(1)) {
    trans <- function(y) -log(y, base)
    inv <- function(y) base^(-y)
    trans_new(paste0("reverselog-", format(base)), trans, inv,
              log_breaks(base = base),
              domain = c(1e-100, Inf))
}

ggplot(data = data_figure4, aes(x = SCOC, y = Depth)) +
    geom_point(alpha = 0.5, size=1.5, shape=1) +
    theme_classic() + xlab(bquote(''*SCOC~(mmol~O[2]~m^-2~d^-1*''))) +
  ylab("Depth (m)") + scale_y_continuous(trans=reverselog_trans(10), breaks =
c(1, 10, 100, 1000, 10000)) + scale_x_continuous(trans=log_trans(10), breaks
= c(1, 10, 100, 1000), position = "top") +
```

```
    theme(axis.text.x = element_text(vjust=0.5, size=15)) +
  theme(axis.text.y = element_text(vjust=0.5, size=15)) +
  theme(text = element_text(size=20)) + annotation_logticks(sides = "tl") +
  theme(legend.title = element_blank()) + guides(col = guide_legend(nrow=3))
+
  theme(legend.position = "none") + theme(plot.margin=unit(c(0.5,0.5,0.5,0.5)
, "cm")) + facet_wrap(~ Ocean, ncol=3)
```

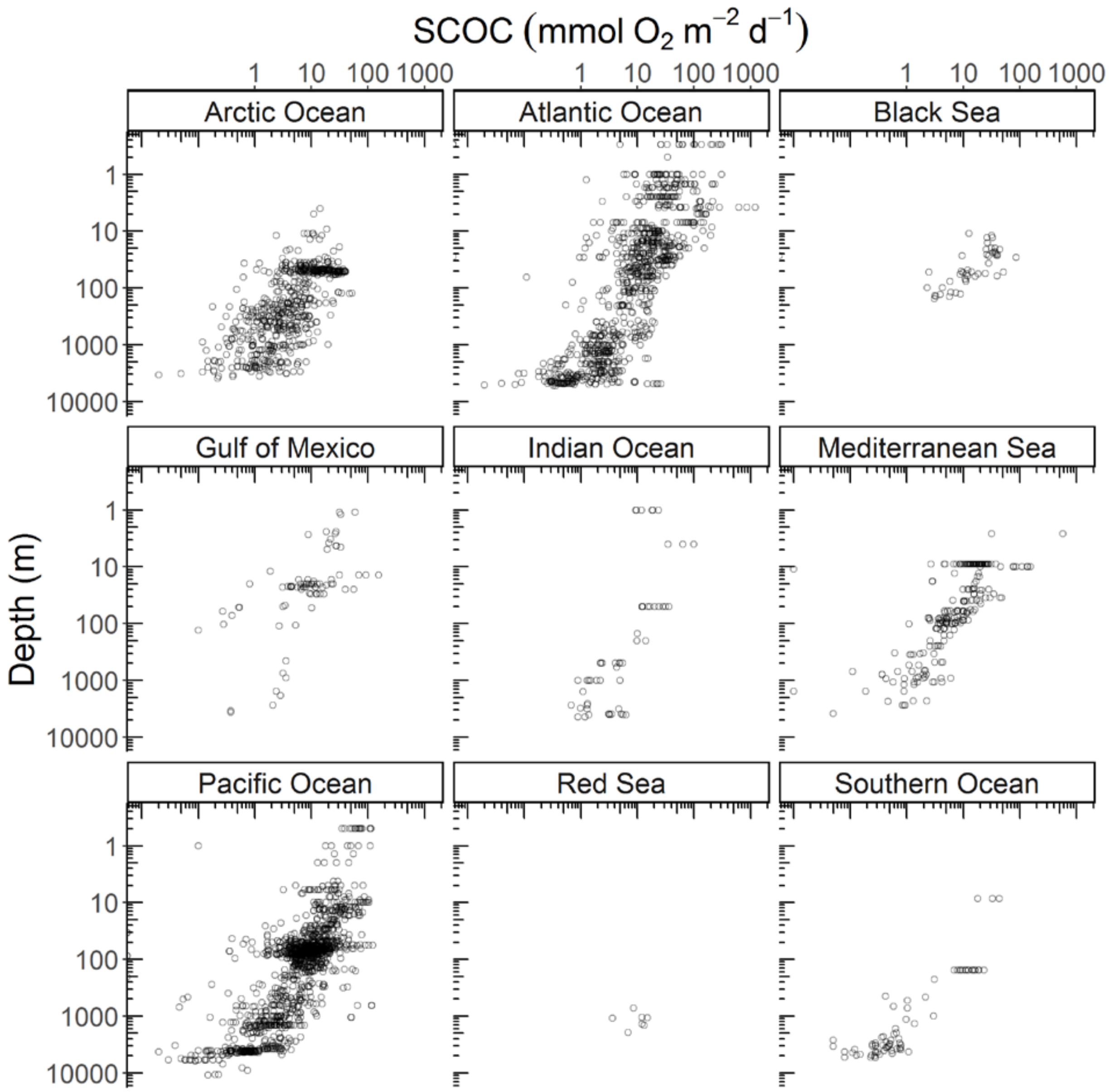


```
ggsave(file="Figure 4.png", width=9,height=9)
ggsave(file="Figure 4.pdf", width=9,height=9)
```
